# Brain-derived neurotrophic factor promotes human granulosa-like tumor cell steroidogenesis and proliferation by activating the FSH receptor-mediated signaling pathway

**DOI:** 10.1038/s41598-017-00203-x

**Published:** 2017-03-15

**Authors:** Min Xie, Meiling Li, Ji Zhou, Xiaomeng Ding, Yidan Shao, Jun Jing, Yuxiu Liu, Bing Yao

**Affiliations:** 1 Center of Reproductive Medicine, Jinling Hospital, Clinical School of Medical College, Nanjing University, Nanjing, Jiangsu People’s Republic of China; 2 Department of Medical Statistics, Jinling Hospital, Nanjing University, Nanjing, Jiangsu People’s Republic of China

## Abstract

Brain-derived neurotrophic factor (BDNF) and FSH receptor (FSHR) are expressed in ovarian granulosa cells, and play important roles in regulating follicle growth and oocyte maturation. Studies have linked the BDNF-associated signaling pathway to FSHR mRNA expression in the regulation of follicle development, but the mechanisms remain unknown. In the current study, we found that BDNF stimulated the secretion of estradiol and progesterone, and increased the proliferation of KGN cells (human granulosa-like tumor cell line). BDNF treatment also increased phosphorylated and ubiquitinated FSHR, and activated cAMP/PKA/CREB signaling pathway. Moreover, inhibition of BDNF expression by siRNA markedly reduced the estradiol secretion and down-regulated FSHR, aromatase and phosphorylated CREB; meanwhile, FSH treatment partly alleviated the effects of BDNF siRNA on KGN cells. These findings suggested that BDNF modulates graunlosa cell functions and the action probably mediated by FSHR-coupled signaling pathway, to affect aromatase-mediated steroidogenesis. These results provide an alternative target to optimize ovarian granulosa cell function.

## Introduction

Brain-derived neurotrophic factor (BDNF) is a member of the neurotrophin family of growth factors^1^ and initiates its biological functions by interacting with a specific Trk receptor tyrosine kinas B (TrkB) or the pan-neurotrophin receptor p75NTR^2^. BDNF is expressed in the nervous system and many peripheral tissues, including the heart, muscle, liver, and reproductive system^3, 4^. In the ovary, BDNF expression was demonstrated in mural and cumulus granulosa cells^5^; it was also detected in the follicular fluid^6^. It is noted that BDNF functions as a regulator of ovarian development, including follicle growth, oocyte maturation and accelerating the extrusion of polar bodies^6^. Evidence indicates that cAMP treatment increases BDNF concentration in granulosa lutein cell lysates, suggesting a potential contribution of BDNF in maintaining the corpus luteum^7^.

Follicle-stimulating hormone receptor (FSHR) is a G protein-coupled receptor (GPCR) consisting of intracellular, transmembrane and extracellular domains^8, 9^; it is predominantly expressed in the ovarian granulosa cells^9^. FSHR plays essential roles in the regulation of steroidogenesis and follicle proliferation during ovary maturation. By increasing the FSHR and aromatase expression, the FSH function in granulosa cells is to convert androgens to estrogens^10^. Besides binding the ligand FSH, the functions of FSHR are modulated by multiple factors. Several mutations affect FSHR’s biological activity, and have been linked to primary amenorrhea, ovarian hyperstimulation syndrome, primary ovarian failure, and infertility^11^. The Ala189Val mutation of the FSHR gene results in a complete blocking of FSH action and failure of human chorionic gonadotropin (hCG) to increase ovarian estradiol secretion^12^. Moreover, FSHR functions can be modulated by post-translational modifications (PTMs), including glycosylation and phosphorylation^13, 14^. Since glycosylation is required for protein folding, glycosylated FSHR facilitates intracellular trafficking for cell surface expression. Besides, phosphorylation occurs after the receptor interacts with its ligand FSH, and is thought to be related to the internalization of the ligand-bond receptor to intracellular sites^15^.

FSH/FSHR-induced signaling is involved in the modulation of various processes related to the steroidogenesis and nuclear events in granulosa cells. Importantly, FSHR is coupled to the classical cAMP/protein kinase A (PKA) signaling pathway^16^, which is a key pathway in the regulation of transcription factors activity^9^. Furthermore, the transcription factor cAMP responsive elements binding protein (CREB) is sufficient to activate the aromatase, a rate-limiting enzyme that regulates steroidogenesis^17^. Moreover, FSHR is also involved in the activation of the PI3K/Akt^18^ and ERK^19^ signaling pathways, which are also involved in the regulation of target genes in granulosa cells. Therefore, by coupling these pathways, the indispensable functions of FSHR in granulosa cells could be performed^20^.

Collectively, the above findings suggest that BDNF may potentially affect granulosa cells through FSHR. To test this hypothesis, we analyzed the BDNF and BDNF siRNA treated KGN cells to explore their effects on FSHR expression and function. The KGN cell line is a steroidogenic human ovarian granulose-like tumor cell line considered a very useful model for researching steroidogenesis, cell growth and FSHR-coupled signaling pathways in human granulosa cells^21^. Moreover, KGN cells secrete estradiol and progesterone, and FSH binding to KGN cells was also demonstrated^21^. Thus, this appropriate cell model was used to explore the mechanisms of BDNF-modulated FSHR and the roles of FSHR-mediated signaling pathways in the regulation of steroidogenesis and proliferation in granulosa cells.

## Results

### KGN cells secrete BDNF and the secretion is enhanced by FSH treatment

**Figure 1:**
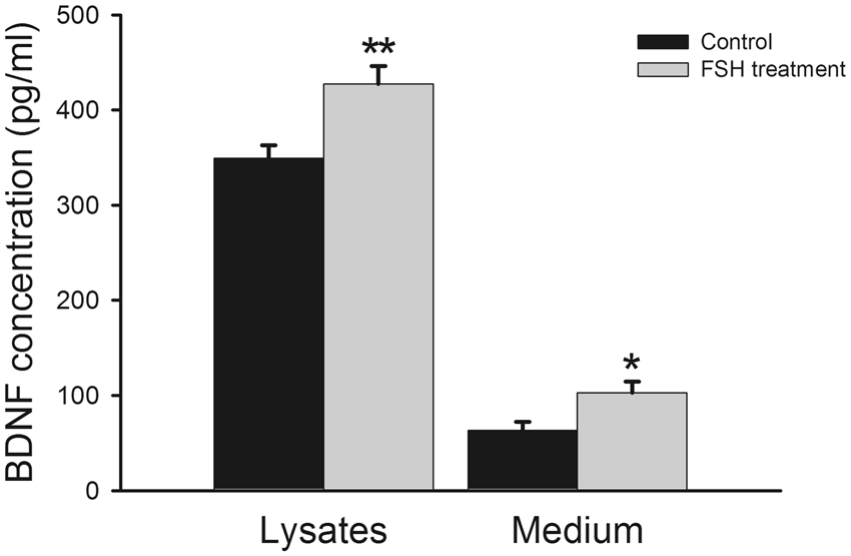
BDNF expression in KGN cells. After treatment (or not) of KGN cells with FSH (100 ng/ml) for 24 h, cell lysates and culture supernatants were collected. Production and secretion of BDNF from FSH treated and untreated cells were detected by ELISA. *P < 0.05 and **P < 0.01 versus control group.

In the current study, we first determined BDNF production in KGN cells by ELISA. BDNF was detected both in lysates (349.3 ± 13.9 pg/ml) and cell culture supernatants (63.2 ± 9.2 pg/ml), suggesting that BDNF was produced and secreted by KGN cells (Fig. 1). Previous research showed that gonadotrophin increased BDNF transcript level of non-stimulated granulosa cells^22^. KGN cells were treated with FSH, and increased BDNF protein level was found in lysates (427.4 ± 18.9 pg/ml) and cell culture supernatants (102.8 ± 11.9 pg/ml) (Fig. 1), indicating that BDNF secretion was stimulated by gonadotrophin. These results demonstrated that KGN cells have common characteristics of normal human granulosa cells, i.e. production and secretion of BDNF.

### Steroidogenesis is promoted by BDNF

**Figure 2:**
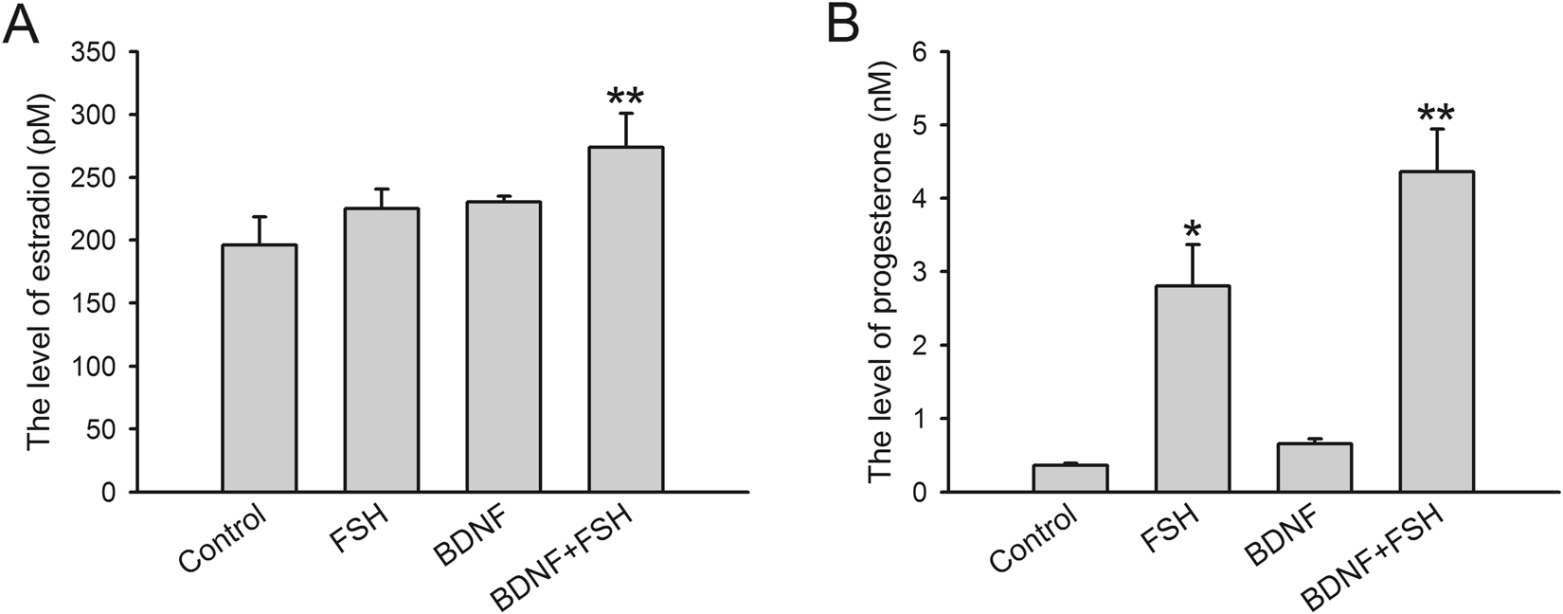
Estradiol and progesterone secretion in KGN cells after treatment. KGN cells were treated with FSH (100 ng/ml), BDNF (5 ng/ml) and FSH (100 ng/ml) plus BDNF (5 ng/ml), respectively, for 24 h. Production and secretion of estradiol (**A**) and progesterone (**B**) were detected by the chemiluminescent immunoassay system. *P < 0.05 and **P < 0.01 versus control group.

Steroidogenesis is one of the major physiological functions of granulosa cells. We next assessed the effects of BDNF treatment on steroidogenesis in KGN cells. The results showed that compared with control group (196 ± 22.5 pM), treatment with FSH (225 ± 15.7 pM) or BDNF (230 ± 4.5 pM) alone had no notable effects on estradiol level, while combined treatment with BDNF and FSH (274 ± 26.9 pM) markedly increased estradiol secretion (Fig. 2A). For progesterone production, compared with control group (0.36 ± 0.03 nM), BDNF alone (0.66 ± 0.07 nM) did not enhance the secretion in granulosa cells, while FSH (2.80 ± 0.57 nM) and BDNF plus FSH (4.36 ± 0.59 nM) both promoted secretion, with higher level obtained for the latter treatment (Fig. 2B). These results suggested that combination of FSH and BDNF significantly elevated hormone secretion in KGN cells.

### BDNF increases KGN cell proliferation

**Figure 3:**
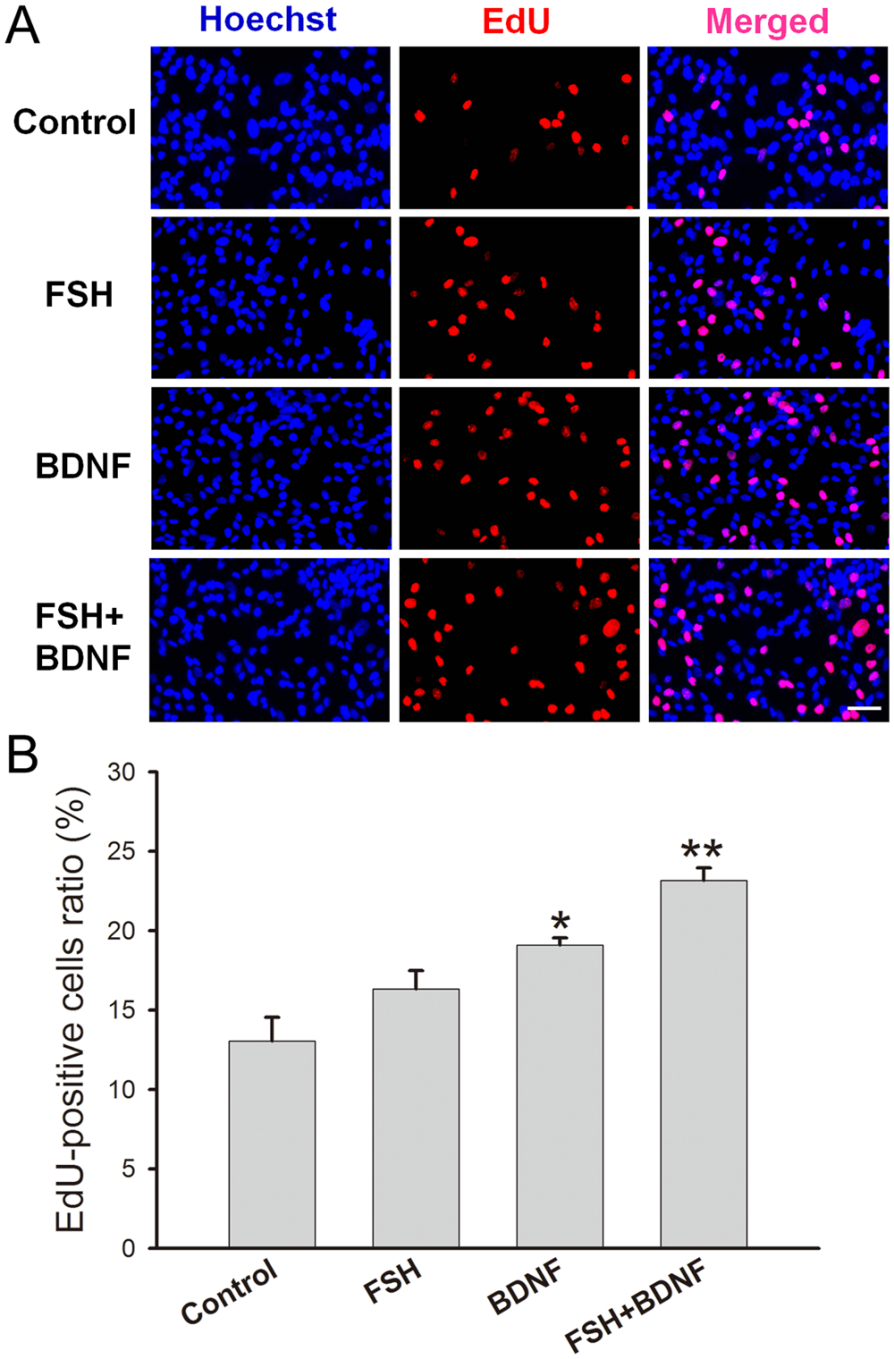
Effect of BDNF on cell proliferation. KGN cells were treated with FSH (100 ng/ml), BDNF (5 ng/ml) and FSH (100 ng/ml) plus BDNF (5 ng/ml), respectively, for 24 h, and cell proliferation was detected by EdU assay. (**A**) Red fluorescence represents EdU-positive cells and blue fluorescence shows cell nuclei. (**B**) Relative ratio of EdU-positive cells of treated and untreated groups. *P < 0.05 and **P < 0.01 versus control group. Scale bar = 50 μm.

Subsequently, the proliferation of KGN cells was evaluated by the EdU cell proliferation assay upon treatment. EdU (5-ethynyl-2′-deoxyuridine) is a nucleoside analog of thymidine, and is incorporated into DNA during active DNA synthesis. Compared with the control group (13.0 ± 1.5%), higher intensity of red fluorescence (EdU) was observed in both BDNF (19.1 ± 0.4%) and FSH plus BDNF treatment group (23.2 ± 1.2%); FSH induced a slight but not significant increase (16.3 ± 0.8%) (Fig. 3A). Quantitation showed that BDNF and combined treatment significantly increased EdU-positive cells (Fig. 3B). These results demonstrated that BDNF promoted cell proliferation, independent of stimulatory factor; moreover, combined treatment facilitated the effect.

### BDNF treatment affects FSHR expression and post-translational modifications

**Figure 4:**
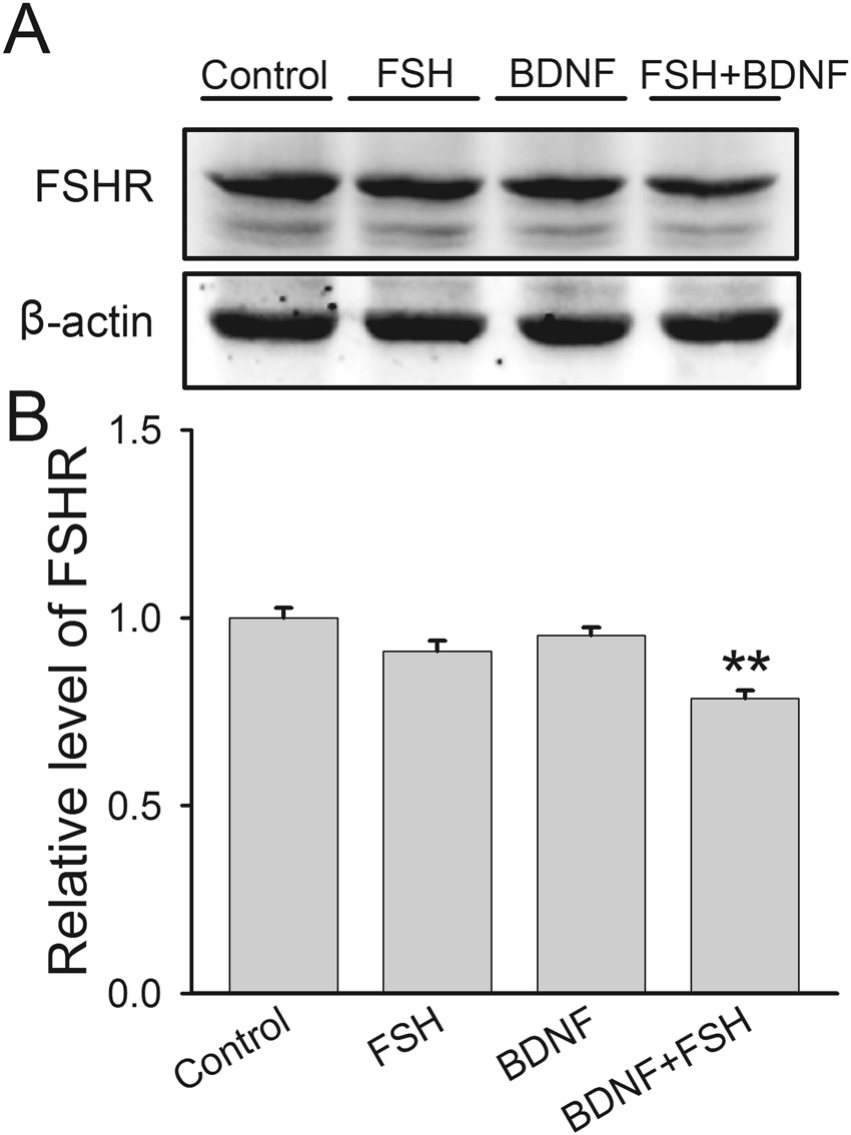
FSH receptor expression in treated KGN cells. KGN cells were treated with FSH (100 ng/ml), BDNF (5 ng/ml) and FSH (100 ng/ml) plus BDNF (5 ng/ml), respectively, for 24 h. (**A**) Western blot analysis of protein extracts from treated and untreated cells, showing FSHR protein level. (**B**) Quantitative analysis, with FSHR protein levels expressed as fold change over controls. Data are mean ± SD. **P < 0.01 versus control group.

**Figure 5:**
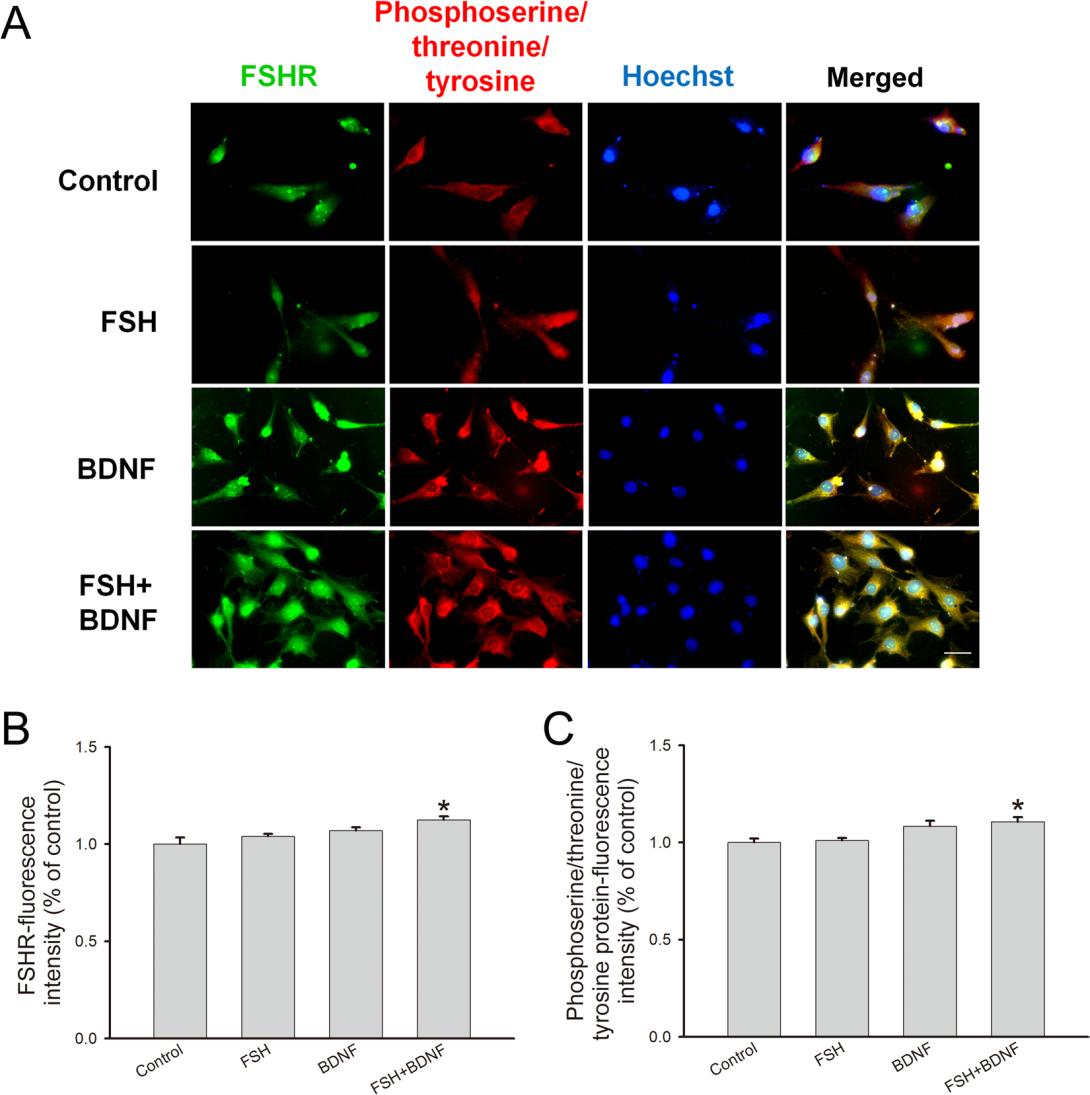
Co-localization of FSH receptor and phosphorylated protein**.** (**A**) After treatment, cells were fixed and subjected to immunofluorescence. Anti-FSHR antibody was probed with anti-rabbit IgG H&L (FITC) (green); antibody against serine, threonine, and tyrosine phosphorylated proteins was probed with anti-mouse IgG H&L (TRITC) (red). Cells were counterstained with Hoechst (blue). Co-localization of FSHR and serine, threonine, and tyrosine phosphorylated proteins was shown in merged images (yellow). (**B**) Quantification of FSHR staining in control and treated cells. (C) Quantification of phosphorylated staining in control and treated cells. *P < 0.05 versus control group. Scale bar = 500 μm.

**Figure 6:**
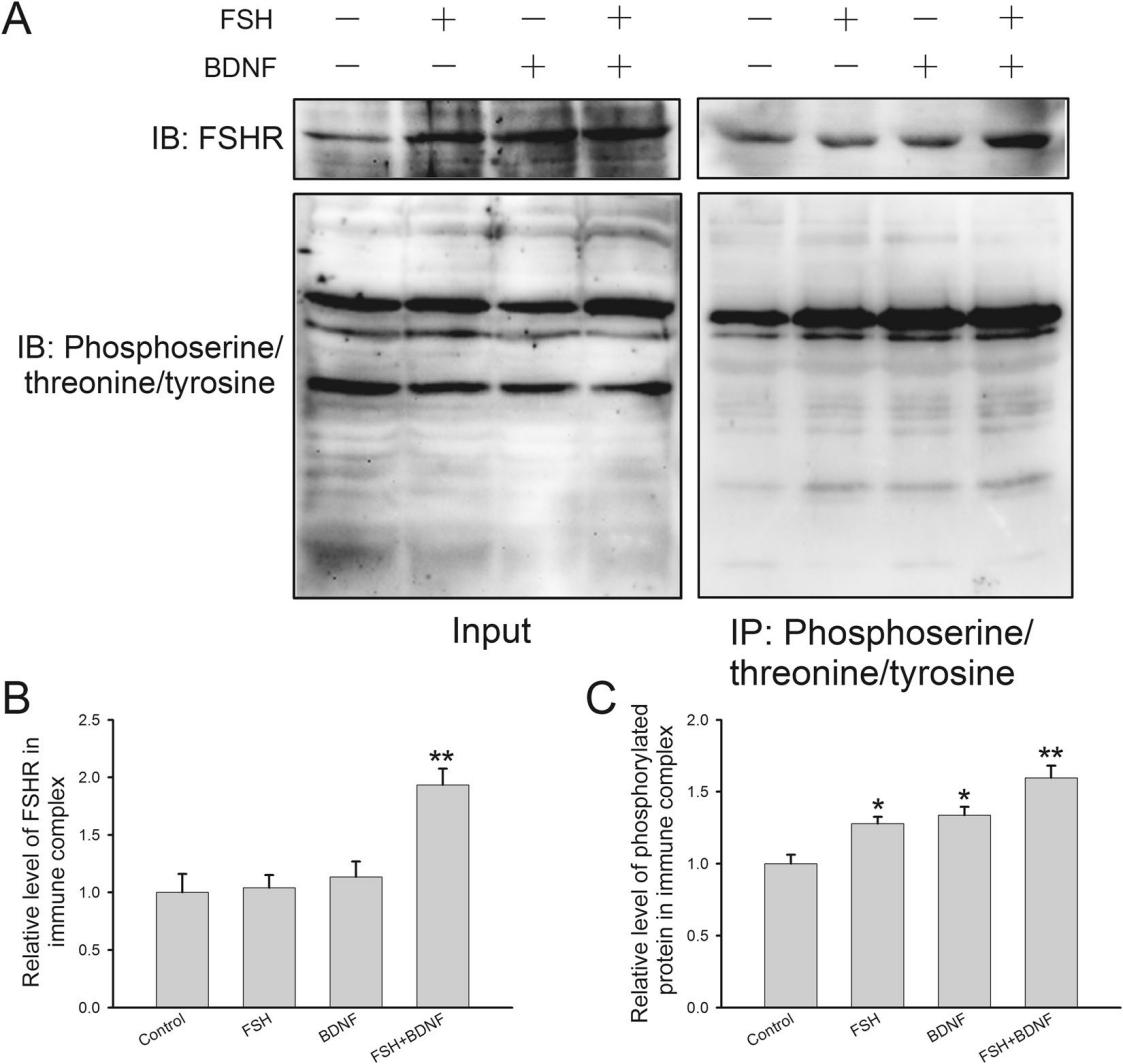
Changes of FSHR phosphorylation in treated KGN cells. (**A**) Cell extracts were immunoprecipitated with anti-phosphoserine/threonine/tyrosine protein antibody and immunoblots were probed with anti-FSHR antibody to detect phosphorylated FSHR. The “input” panel shows target proteins prior to immunoprecipitation in the extracts. (**B,C**) Quantitative analysis showing the levels of the FSHR protein and phosphorylated protein in immune complexes. Protein level is expressed as fold change over control; data are mean ± SD. *P < 0.05 and **P < 0.01 versus control group.

**Figure 7:**
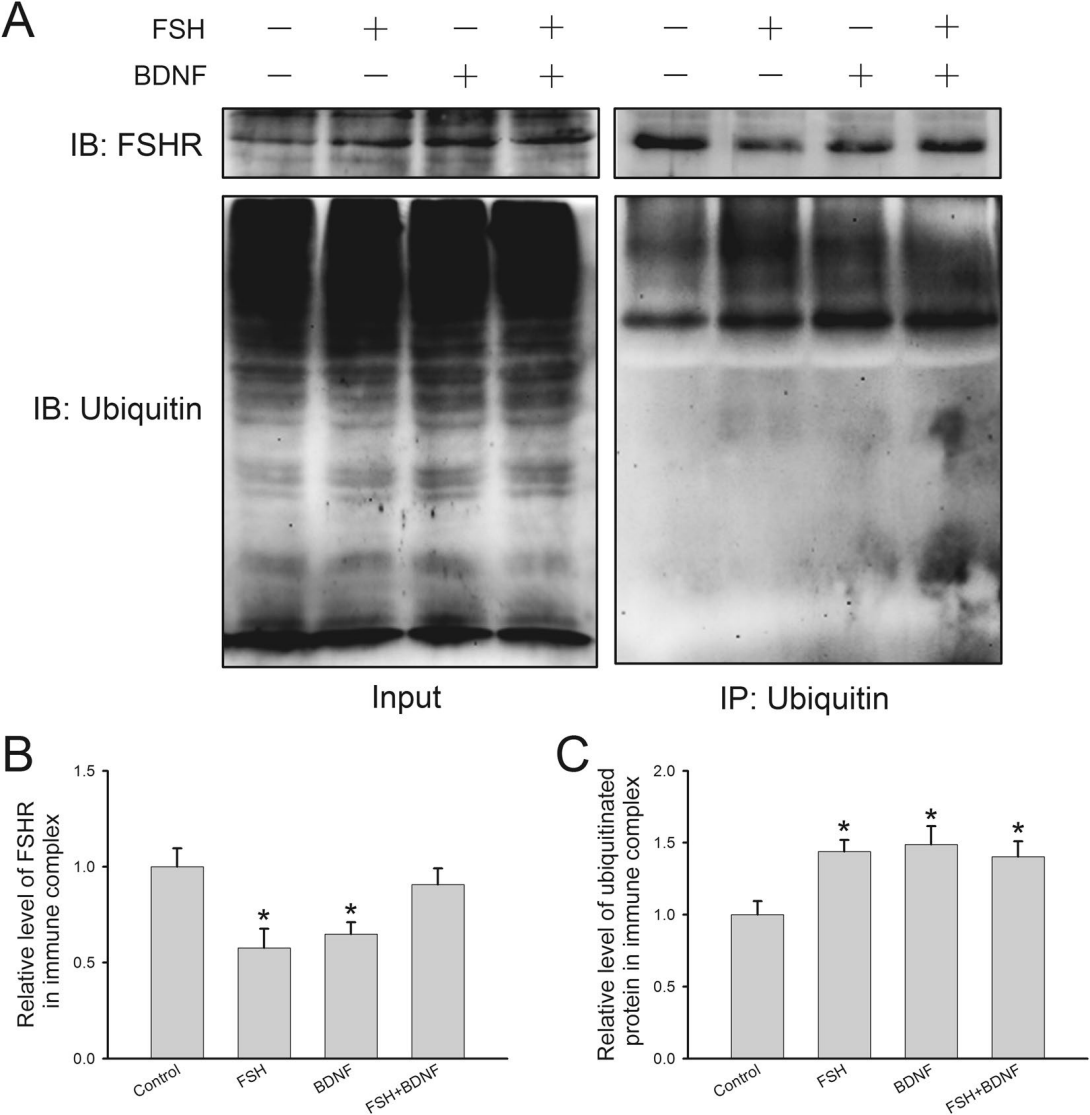
Changes of FSHR ubiquitination in treated KGN cells. (**A**) Cell extracts were immunoprecipitated with anti-ubiquitin antibody, and immunoblots were probed with anti-FSHR antibody to detect ubiquitinated FSHR. The “input” panel shows target proteins prior to immunoprecipitation in the extracts. (**B,C**) Quantitative analysis showing the levels of FSHR protein and ubiquitinated protein in immune complexes. Protein level is expressed as fold changes over control; data are mean ± SD. *P < 0.05 versus control group.

Since the secretion of hormones is intimately associated with the activation of FSHR-mediated pathways, we hypothesized that BDNF probably had an effect on FSHR expression and modifications that play essential roles in the regulation of FSHR functions. First, decreased FSHR protein level was observed after BDNF plus FSH treatment (0.78 ± 0.02), while BDNF (0.95 ± 0.02) or FSH (0.91 ± 0.02) alone had no significant effect (Fig. 4). Secondly, we measured FSHR phosphorylation by double immunofluorescence staining, and found a co-localization of FSHR with the phosphorylated protein (Fig. 5A). Furthermore, compared with the control group, signal intensities of FSHR (FSH, 1.04 ± 0.01; BDNF, 1.07 ± 0.02; BDNF + FSH, 1.12 ± 0.02) and phosphorylated protein (FSH, 1.01 ± 0.01; BDNF, 1.08 ± 0.03; FSH + BDNF, 1.11 ± 0.02) were both increased by BDNF plus FSH treatment (Fig. 5B and C). Finally, immunoprecipitation was used to detect FSHR binding with phosphorylated or ubiquitinated proteins. In Fig. 6, input represents a positive control to demonstrate the existence of phosphorylated proteins and FSHR in the cell extracts. Immune complexes were obtained from cell lysates after incubation with anti-phosphoserine/threonine/tyrosine antibody. The presence of FHSR in the immunoprecipitates was detected by probing with anti-FSHR antibody. FSHR was detected in immune complexes; and treatment with FSH plus BDNF increased the association of FSHR (control, 1.00 ± 0.16; FSH, 1.04 ± 0.11; BDNF, 1.13 ± 0.14; FSH ± BDNF, 1.93 ± 0.14) with phosphorylated protein (control, 1.00 ± 0.06; FSH, 1.28 ± 0.05; BDNF, 1.34 ± 0.06; FSH ± BDNF, 1.60 ± 0.08) significantly (Fig. 6), demonstrating that treatment could increase phosphorylated FSHR. For FSHR ubiquitination, compared with the control group, BDNF or FSH treatment decreased the ubiquitinated FSHR in immune complexes, while combination treatment increased FSHR association with ubiquitinated protein (IP-FSHR: control, 1.00 ± 0.10; FSH, 0.58 ± 0.10; BDNF, 0.65 ± 0.06; FSH + BDNF, 0.91 ± 0.08 and IP-ubiquitinated protein: control, 1.00 ± 0.10; FSH, 1.44 ± 0.08; BDNF, 1.49 ± 0.12; FSH + BDNF, 1.40 ± 0.11) (Fig. 7). Collectively, these findings suggested that BDNF plus FSH treatment decreased FSHR level and enhanced FSHR phosphorylation and ubiquitination.

### BDNF treatment activates the cAMP/PKA/CREB pathway

**Figure 8:**
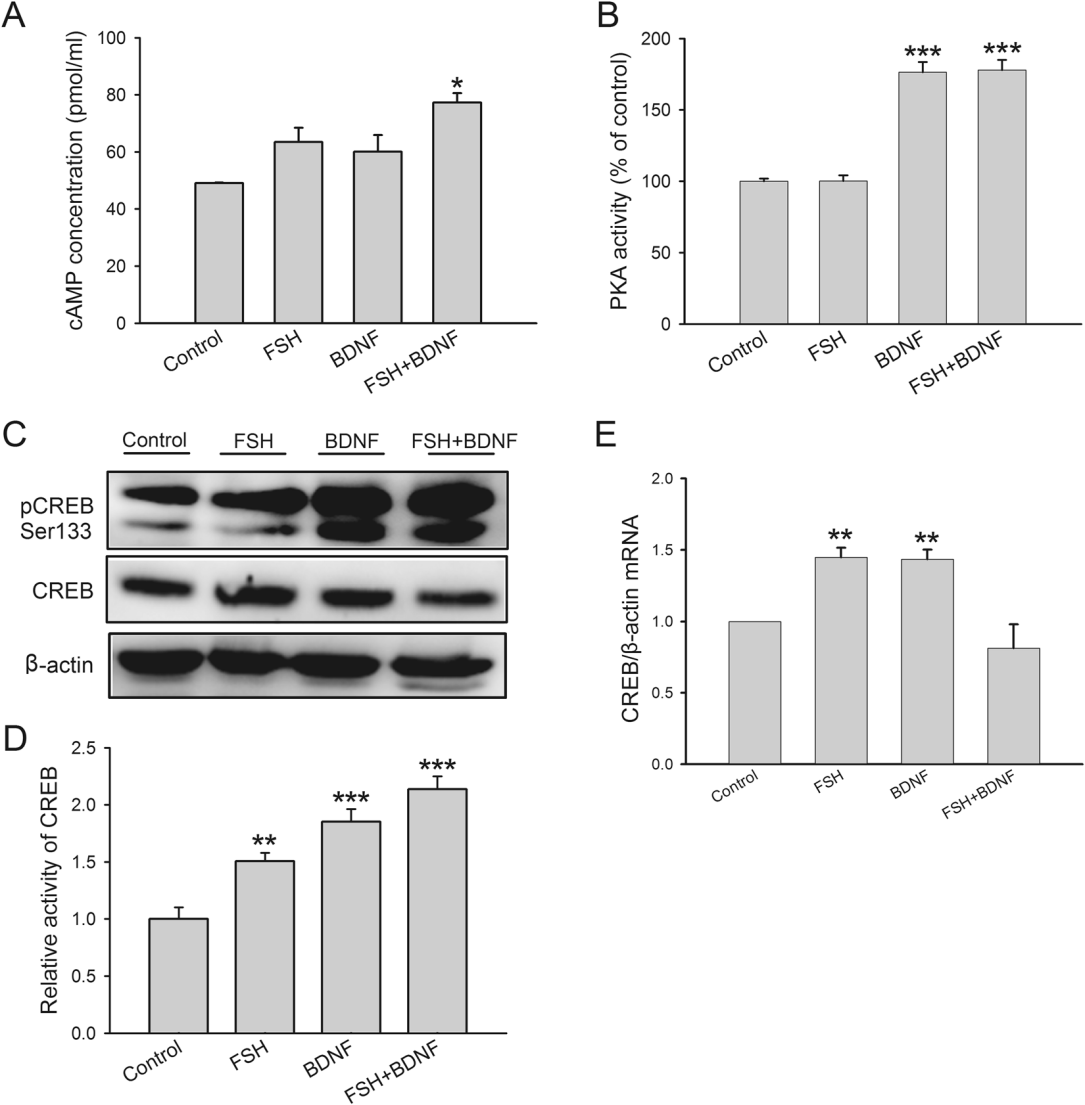
Effects of BDNF on cAMP level, PKA and CREB activities, and CREB mRNA expression. KGN cells were treated with FSH (100 ng/ml), BDNF (5 ng/ml) and FSH (100 ng/ml) plus BDNF (5 ng/ml), respectively, for 24 h. (**A** and **B**) Cell extracts were subjected to ELISA for the determination of cAMP production and PKA kinase activity in treated and untreated cells. (**C**) Western blot analysis of protein extracts from treated and untreated cell showing the levels of pCREB (Ser133) and total CREB. (**D**) Relative CREB activity was derived as pCREB normalized to total CREB. (**E**) Total cellular RNA was extracted, and CREB mRNA levels were determined by real-time PCR. Relative gene expression levels were normalized to β-actin, and expressed as fold change. *P < 0.05, **P < 0.01 and ***P < 0.001 versus control group.

Since FSHR is associated with the cAMP/PKA signaling pathway, and activated PKA phosphorylates CREB, we assessed whether cAMP/PKA/CREB signaling pathway was activated by BDNF. First, ELISA was used to determine cAMP production and PKA activity. Accumulation of cAMP was elicited by FSH, BDNF and FSH plus BDNF stimulation and significant difference was detected in combined treatment (control, 49.1 ± 0.2 pmol/ml; FSH, 63.5 ± 5.0 pmol/ml; BDNF, 60.1 ± 5.9 pmol/ml; FSH + BDNF, 77.3 ± 3.3 pmol/ml) (Fig. 8A). Secondly, the PKA kinase activity assay showed that both BDNF and combined treatment induced remarkable elevation of PKA activity (control, 100.0 ± 1.8%; FSH, 100.2 ± 4.0%; BDNF, 176.3 ± 7.3%; FSH + BDNF, 178.0 ± 7.1%) (Fig. 8B). PKA activation phosphorylated CREB and initiated its transcription. In addition, CREB phosphorylation at Ser133 is central to the activation of CREB-mediated gene transcription^23, 24^. Although treatment with FSH, BDNF, and FSH plus BDNF increased the phosphorylated CREB (Ser133), increasing CREB activity notably whiles the BDNF existence (control, 1.00 ± 0.10; FSH, 1.51 ± 0.07; BDNF, 1.85 ± 0.11; FSH + BDNF, 2.14 ± 0.11) (Fig. 8C,D). We also assessed CREB mRNA levels, and found increased CREB mRNA upon FSH or BDNF treatment in comparison with control group, but the combined treatment did not induce such effect (control, 1.00 ± 0.00; FSH, 1.44 ± 0.07; BDNF, 1.43 ± 0.07; FSH + BDNF, 0.81 ± 0.16) (Fig. 8E). Collectively, these findings demonstrated that BDNF induced the activation of the cAMP/PKA/CREB signaling pathways.

### BDNF suppression down-regulates estradiol level and inhibits steroidogenesis, effects alleviated by FSH induction

**Figure 9:**
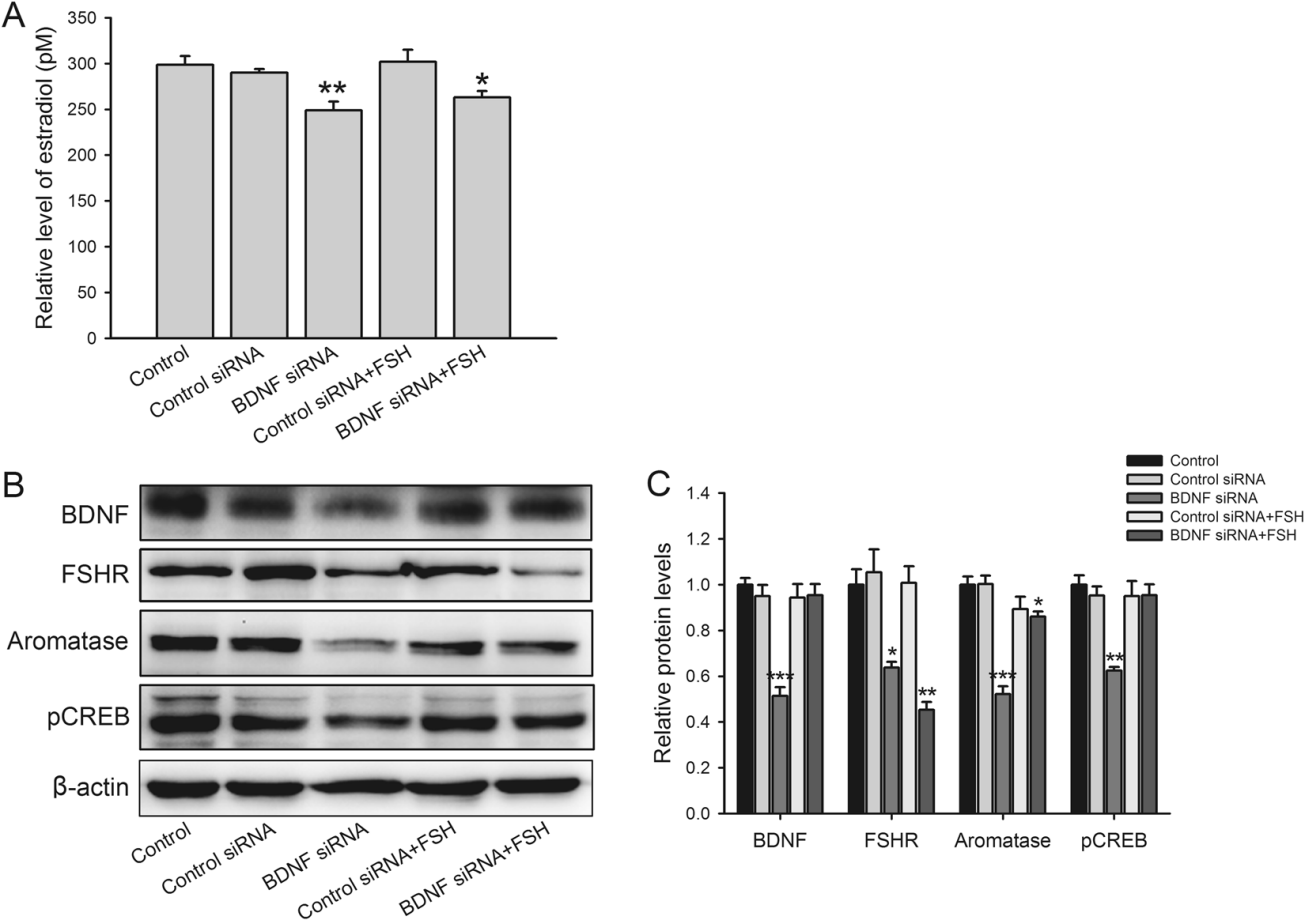
Effects of BDNF siRNA and FSH treatment on estradiol secretion, and FSHR, aromatase and phosphorylated CREB levels. (**A**) Production and secretion of estradiol was detected by chemiluminescent immunoassay system. (**B**) Western blotting measured the protein levels of BDNF, FSHR, aromatase and pCREB (Ser133) in KGN cells transfected with BDNF siRNA. (**C**) Relative protein levels in (**B**) are expressed as fold changes over control. *P < 0.05, **P < 0.01 and ***P < 0.001 versus control group.

As the BDNF plus FSH treatment affected steroidogenesis (Fig. 2) and FSHR expression (Fig. 4A), we assessed whether BDNF knockdown may play a crucial role in the regulation of steroidogenesis. To test this hypothesis, siRNA targeting the *BDNF* gene was used to silence its expression. Compared with the control group (298.6 ± 9.5 pM), BDNF knockdown significantly decreased estradiol secretion (control siRNA, 290 ± 4.0 pM; BDNF siRNA, 249 ± 9.5 pM), and FSH partly rescued the production of estradiol (control siRNA + FSH, 302 ± 13.1 pM; BDNF siRNA + FSH, 263 ± 7.0 pM) (Fig. 9A). In addition, decreased levels of BDNF (control siRNA, 0.95 ± 0.05; BDNF siRNA, 0.51 ± 0.04), FSHR (control siRNA, 1.05 ± 0.10; BDNF siRNA, 0.64 ± 0.02), aromatase (control siRNA, 1.00 ± 0.04; BDNF siRNA, 0.52 ± 0.03) and phosphorylated CREB at Ser133 (control siRNA, 0.95 ± 0.04; BDNF siRNA, 0.62 ± 0.02) were observed, and the inhibitory effects of BDNF siRNA were partly rescued by FSH treatment (Fig. 6B,C), especial aromatase (control siRNA + FSH, 0.89 ± 0.05; BDNF siRNA + FSH, 0.86 ± 0.02 pM) and phosphorylated CREB (control siRNA + FSH, 0.95 ± 0.06; BDNF siRNA + FSH, 0.95 ± 0.05), which suggested FSH alleviated steroidogenesis pathway inhibition induced by BDNF deficiency.

## Discussion

It is well known that ovarian BDNF functions as a regulator of follicular development and oocyte maturation^25, 26^. In the current study, we demonstrated that KGN cells produced BDNF, and FSH treatment increased this production. Furthermore, BDNF treatment and BDNF knockdown were used to determine how BDNF affects the expression and modifications of FSHR, as well as the activities of FSHR-coupled cAMP/PKA/CREB signaling pathway. Through these explorations, the molecular mechanisms underlying the effects of BNDF on hormone secretion were elaborated.

BDNF is critical to the regulation of cell function and survival. The BDNF protein is first synthesized as a precursor protein, proBDNF, which is then cleaved to generate mature BDNF (mBDNF); only mBDNF is considered to be biologically active. There are probably two secretory pathways of proBDNF^27^. It was proposed that proBDNF processing into mBDNF occurs extracellularly, and is dependent on the extracellular proteases, as found in cultured hippocampal neurons^28^. Yang *et al*. also indicated that proBDNF is secreted^29^. An alternative pattern was proposed that proBDNF produced by neurons is rapidly converted to mBDNF intracellularly for storage, and released by excitatory input^30^. According to the above studies, whether proBDNF cleavage occurrs intracellularly or extracellularly, mBDNF has extracellular biological functions. Therefore, in the current study, BDNF was added in the medium was to increase extracellular BDNF directly, while BDNF knockdown by siRNA aimed to decrease BDNF secretion.

Estradiol and progesterone are major hormones that regulate ovarian growth and physiological function^31–34^. In mammals, circulating estrogens are mainly produced by the granulosa cells, and required for the development of secondary sex characters in females^35^. Estradiol mainly enhances the response of granulosa cells to gonadotropins^36^ and plays essential roles in follicular development^37^. Progesterone secretion is critical for ovulation, as well as establishment and maintenance of pregnancy. According to the classic “two-cell, two-gonadotropin” model, estradiol is transformed from androgens which is synthesized from progesterone^38^. Our data showed a dramatically increased estradiol and progesterone after combined treatment with BDNF and FSH (Fig. 2), suggesting synergistic effects of BDNF and FSH in inducing hormone secretion in KGN cells. Accordingly, increased estradiol and progesterone levels induced by BDNF and FSH (Fig. 2) provided alternative options for promoting steroidogenesis in granulosa cells.

Besides promoting steroidogenesis, BDNF also increased KGN cell proliferation. We found a statistically significant increase in EdU-positive cells upon BDNF and BDNF plus FSH treatments (Fig. 3), which indicated the inductive effect of BDNF on granulosa cells proliferation. Similar biological effect was observed in the central nervous system. BDNF was shown to stimulate the differentiation of neural progenitor cells, regulating neurogenesis^39^. Moreover, BDNF administration or its increased expression promotes hippocampal neurogenesis and increases neuronal activity^40, 41^. Therefore, BDNF not only regulates the neurons, but also promotes growth and physiological functions (steroidogenesis) in granulosa cells.

To elicit ovarian steroidogenesis, functional FSHR is required^16^. Our findings indicated FSHR protein level decreased by combined treatment (Fig. 4). FSHR activity is regulated by multiple patterns, including mutations and post-translational modifications^20^. FSHR mutation in female animals induces ovarian dysfunction, including smaller ovaries, and no corpora lutea and mature Graafian follicles, suggesting that FSHR mutation could block follicular maturation^42^. In addition, post-translational modifications of the FSHR protein, such as phosphorylation, have pivotal roles in a variety of processes^43^. Receptor phosphorylation commonly occurs on serine and threonine, and is linked to FSHR desensitization and internalization^14^. Desensitization is an important physiological feedback mechanism that protects against receptor overstimulation. GPCR desensitization also plays essential roles in integrating biological signals through second messenger protein kinase-dependent phosphorylation^44^. Desensitization and internalization are prominent to the GPCR in the regulating downstream signaling events and recycling back to the cell surface^44, 45^. It is thought that after internalization, one of the optional locations of the receptor is the lysosome^45^. However, another degradation pathway has been proposed, namely the ubiquitin-proteasome pathway (UPP). Previous findings demonstrated that FSHR can be ubiquitinated and degraded by the proteasome, since treatment with the proteasome inhibitor MG132 increases FSHR level^46^. Accordingly, decreased FSHR protein level, and increased phosphorylation and ubiquitination of FSHR induced by BDNF plus FSH treatment (Figs 4 to 7) suggested that BDNF probably facilitates FSHR internalization and degradation of (might be mainly through UPP), by inducing its phosphorylation and ubiquitination.

PTMs act as molecular switches and initiate protein-protein interactions. Protein degradation can be controlled by PTM crosstalk, e.g. phosphodegrons in ubiquitin-mediated protein degradation^43^. FSHR phosphorylation and ubiquitination have been reported, but the association of the two modifications remains largely unclear. Our findings indicated that BDNF affects FSHR expression, and promotes its phosphorylation and ubiquitination. Phosphorylation can serve as a signal for recognition by a binding protein (ubiquitin) that carries out ubiquitination^47, 48^. There are many parallels between phosphorylation and ubiquitination. Phosphoproteomic analysis indicates that the majority of proteins in a cell can be phosphorylated, and the number of proteins known to be subject to ubiquitination is steadily increasing^48^. Therefore, increased phosphorylation of FSHR may elicit ubiquitination.

As shown above, increased activity of cAMP/PKA/CREB signaling pathway was induced by BDNF or BDNF plus FSH (Fig. 8). However, FSH alone did not induce the increase cAMP level and PKA activity. CREB phosphorylation at Ser133 is regulated by PKA and other kinases^49^. Nevertheless, CREB is directly phosphorylated by PKA in granulosa cells, not by other kinases^17^. The transcription factor CREB is crucial for stimulus-transcription coupling, which converts the events occuring at cell membrane into an intercellular gene expression; in turn, this ultimately affects the function of individual cells by regulating the expression of certain proteins^49^. CREB regulates genes expression by binding to the cAMP-response element (CRE), a specialized stretch of DNA that within the regulatory region of numerous genes^23^. A point mutation in the DNA-binding domain prevents CREB from binding to CRE, and makes it unable to bind to DNA-binding sites^23^. Thus, the transcriptional activity relies on the combination of CREB and CRE. Analysis of CRE-mediated transcription in KGN cells demonstrated that CRE is essential for aromatase activity^50^.

To further assess the effects of BDNF on hormones biosynthesis and related mechanisms in granulosa cells, BDNF siRNA was used to knockdown BDNF, and down-regulation of estradiol, FSHR, aromatase, and phosphorylated CREB were observed (Fig. 9). Aromatase is an enzyme responsible for a key step in estrogens biosynthesis. FSHR mediates the activation of the cAMP/PKA signaling pathways and subsequently stimulates aromatase expression^51, 52^. Therefore, by triggering cAMP dependent signaling cascades, *CYP19A1* transcription is upregulate, which consequently induces estrogen biosynthesis^52^. In addition, *CYP19A1* transcription is promoted by CREB^53^. Accordingly, the increased estradiol secretion induced by treatment with BDNF plus FSH (Fig. 2) could be a stimulatory factor for cAMP/PKA/CREB signaling pathway, which mediated by FSHR.

In conclusion, this study demonstrated that KGN cells produced BDNF, and treatment with BDNF plus FSH induced the steroidogenesis and KGN cell proliferation. In addition, FSHR modifications and the FSHR-coupled signaling pathway were also regulated by BDNF and BDNF plus FSH. Moreover, the above effects of BDNF could be inhibited by BDNF siRNA. These findings concerning in KGN cells may be applied for a further understanding of the functional regulation of granulosa cells. Overall, this study has established a molecular link between BDNF and FSHR function, partly revealing the mechanisms by which neurotrophic factors modulate hormone secretionin granulosa cells.

## Methods

### Reagents and antibodies

Recombinant human BDNF was purchased from PeproTech (Rocky Hill, NJ, USA). FSH (human pituitary) and protein G agarose were from Merck Millipore (Darmstadt, Germany). Hoechst 33342 for DNA staining was obtained from Sigma-Aldrich (Darmstadt, Germany). The following antibodies were used: rabbit polyclonal antibodies against β-actin, FSHR and BDNF (Bioworld Technology, Louis Park, MN, USA); rabbit monoclonal antibodies against CREB and phospho-CREB (Ser133) (Cell signaling Technology, Danvers, MA, USA); rabbit polyclonal antibody against ubiquitin and aromatase, and mouse monoclonal antibody against phosphoserine/threonine/tyrosine (Abcam, Shanghai, CHN). The following kits were employed: Human BDNF ELISA Kit (Boster, Wuhan, CHN); cAMP assay kit (R&D Systems, Inc., Minneapolis, MN, USA); PKA kinase activity kit (Abcam, Shanghai, CHN).

### KGN cell culture and treatment

KGN cells were seeded in culture dishes and cultured in the DMEM/F12 medium supplemented with 10% fetal bovine serum (Gibco, Thermo Fisher Scientific, USA), penicillin (100 U/ml) and streptomycin (100 μg/ml) in a humidified incubator containing 5% CO_2_ at 37 °C. Four cell groups were set up: control (PBS); FSH (FSH, 100 ng/ml); BDNF (BDNF, 5 ng/ml); FSH and BDNF (FSH at 100 ng/ml and BDNF at 5 ng/ml) treatment. After 24 h of incubation, the culture medium and whole-cell extracts were obtained for biochemical analyses.

### Hormone Assays

After treatment, the culture medium from control and treated cells was collected and centrifuged at 1000 g for 3 min, and supernatants were moved to fresh tubes for the assay. The concentrations of estradiol and progesterone were determined on a chemiluminescent immunoassay system (Backman Coulter, Inc., Brea CA, USA). The intra-assay and inter-assay coefficients of variation for estradiol and progesterone were 5.13% and 6.23%, and 8.18% and 7.89% respectively.

### EdU assay

The effect of BDNF on cell proliferation was assessed with the Cell-Light EdU DNA cell proliferation kit (RiboBio, Guangzhou, CHN), according to the manufacturer’s instructions. Briefly, KGN cells (4 × 10^4^ cells per well) were seeded in 96-well plates. Then, the cells were incubated with 50 μM of EdU for 2 h at 37 °C. After two washes with PBS, the cells were fixed with 4% formaldehyde for 30 min at room temperature. Next, the cells were treated with 0.5% Triton X-100 for 10 min at room temperature and incubated with 1 × Apollo reaction cocktail (100 μl/well) for 30 min. After two washes with 0.5% Triton X-100 for 10 min at room temperature, DNA was stained with Hoechst 33342 (100 μl/well) for 30 min and visualized by fluorescence microscopy. EdU-positive cells were obtained from fluorescent images; confluent cells were randomly selected from each analysis. Cells were counted with ImageJ, and the relative positive ratios were averaged from four values per group.

### ELISA for assessing BDNF and cAMP levels, and PKA kinase activity

BDNF and cAMP levels in cell lysates and culture supernatants were determined using commercially available kits, according to the manufacturer’s protocols in triplicate. Briefly, BDNF levels in cell lysates were adjusted to the amount of protein in the corresponding culture well (pg BDNF/mg protein). Cell culture supernatants and lysates were added to a plate that pre-coated with human BDNF specific antibody, and incubated at 37 °C for 90 min. This was followed by incubation with biotinylated anti-human BDNF antibody at 37 °C for 60 min. After addition of avidin-biotin-peroxidase-complex for 30 min at 37 °C and color development, absorbance was measured at 450 nm on a multi-mode microplate reader (Synergy HTX, BioTek Instruments, INC., USA). The BDNF assay had a linear over a range of 31.2–2000 pg/ml.

For cAMP assay, the primary antibody solution was added for 1 h; then, cAMP conjugate and lysates were added to the assay plate; cAMP levels were measured accoding to the manufacturer’s recommendations at 450 nm on a multi-mode microplate reader. The number of moles of cAMP was calculated by comparing with a curve derived from the standards provided with the kit. The mean minimum detectable dose of cAMP was 1.5 pmol/ml.

For PKA kinase activity evaluation, the wells were soaked with kinase assay dilution buffer for 10 min at room temperature. Then, standards and KGN cell lysates were added to the wells with ATP to initiate the reaction. After incubation at 30 °C for 90 min, the wells were emptied to terminate the kinase reaction, and the phosphospecific substrate antibody was added for 60 min at room temperature. HRP conjugated anti-rabbit IgG was added at room temperature for 30 min prior to a wash step. Subsequently, the TMB substrate solution was added at room temperature for 30–60 min. OD of each well was immediately determined at 450 nm.

### Western blotting analysis

After treatment, the cells were lysed in ice-cold cell lysis buffer (20 mM Tris-HCl [pH 7.5], 150 mM NaCl, 1 mM Na_2_EDTA, 1 mM EGTA, 1% Triton, 2.5 mM sodium pyrophosphate, 1 mM β-glycerophosphate, 1 mM Na_3_VO_4_, 1 μg/ml leupeptin) supplemented with the protease inhibitors aprotinin (2 μg/ml) and phenylmethysulfonyl fluoride (PMSF, 1 mM). Protein concentration was determined by BCA (Pierce, Thermo Fisher Scientific Inc., Rockford, IL, USA). Equal amounts of protein were separated by 10% SDS-PAGE, and electrically transferred onto nitrocellulose membrane (Millipore, Billerica, MA, USA). Subsequently, the membranes were blocked with 5% defatted milk in TBS-T (10 mM Tris-HCl, 150 mM NaCl, 0.1% (v/v) Tween-20, pH 7.5) for 60 min at room temperature. Membranes were then probed with the primary antibodies diluted with 5% BSA and incubated overnight at 4 °C. The following primary antibodies were used: anti-β-actin (1:2000), anti-FSHR (1:500), anti-CREB (1:500), anti-phospho-CREB (Ser133) (1:500), anti-BDNF (1:500), and anti-aromatase (1:1000). The membranes were rinsed and incubated with goat anti-rabbit IgG (H&L)-HRP (1:50,000) for 1 h at room temperature, and visualized on Tanon-5200 (Shanghai, CHN).

### Immunofluorescence

Sterilized coverslips were placed in 24-well plates before seeding the KGN cells, which were cultured in a 5% CO_2_ incubator at 37 °C. At 40–70% confluency, cells were fixed. Then, the cells were rinsed with PBS, fixed with methanol at −20 °C for 8 min, and permeabilized with 0.5% Triton X-100 in PBS buffer for 15 min. After rinsing the cells with 0.2% Triton X-100 in PBS, blocking buffer (5% BSA) was added for 60 min. Primary antibodies, including rabbit anti-FSHR (1:50) and mouse anti-phosphoserine/threonine/tyrosine (1:100), were added for 24 h at 4 °C. After three washes, the cells were incubated with goat anti-rabbit IgG H&L (FITC) and anti-mouse IgG H&L (TRITC) secondary antibodies (Abcam) conjugated to FSHR and phosphoserine/threonine/tyrosine, respectively, at room temperature for 60 min. Subsequently, the cells were washed and counterstained with Hoechst (1 μg/ml) for 15 min. Finally, the cells were mounted with buffered glycerol. Images were acquired by fluorescence microscopy on an IX73 microscope (Olympus Corporation, Shinjuku, Tokyo, JPN). The intensity was analyzed with ImageJ, and the relative fluorescence intensities were averaged from three values per group.

### Immunoprecipitation

After treatment, cells were lysed in ice-cold radio-immunoprecipitation assay (RIPA) buffer (50 mM Tris-HCl [pH 8.0], 150 mM sodium chloride, 1% IGEPAL CA-630 (NP-40), 0.5% sodium deoxycholate, and 0.1% sodium dodecyl sulfate), supplemented with the protease inhibitors aprotinin (2 μg/ml) and PMSF (1 mM) for 20 min, and centrifuged at 12,000 rpm and 4 °C for 20 min. To reduce nonspecific binding, protein G agarose was added to the cell lysates (1:10) and incubated at 4 °C for 30–60 min. This was followed by centrifugation at 12,000 rpm and 4 °C for 15 min, and supernatants were transferred to fresh tubes. Anti-ubiquitin (1:100) or anti-phosphoserine/threonine/tyrosine (1:100) antibodies were then add overnight at 4 °C. Afterward, protein G agarose (1:5) was added to capture immune complexes. After centrifugation for 1 min at 12,000 rpm and 4 °C, the pellet was washed three times with ice-cold PBS. This pellet containing protein G agarose (antibody-binding protein) was resuspended in sample buffer, heated to 95–100 °C for 2–5 min, and centrifuged for 1 min at 12,000 rpm. The resulting solution was then analyzed by Western blotting.

### RNA extraction and quantitative real-time PCR

The treated cells were washed with ice-cold PBS, and incubated with TRIzol reagent (Invitrogen, Life Technologies Ltd, Renfrew, Strathclyde, UK). The cells were collected in RNase free tubes and mixed with chloroform (200 μl/ml TRIzol). After centrifugation for 10 min at 12,000 rpm, an equal volume of isopropanol was added to the supernatant and centrifuged at 12,000 rpm for 10 min. After removing the supernatant, 75% ethanol was add, and the mixture centrifuged at 12,000 rpm for 10 min. The resulting pellet was dissolved with RNase free water. RNA concentrations were adjusted based on absorbance at 260 nm. Reverse transcription was performed in 20 μl reaction volumes with 5 × Prime Script RT Master Mix (Takara, Shiga, JPN, 2 μl), total RNA (1000 ng) and RNase free water at the following conditions: 37 °C for 15 min, 5 °C for 5 s, and 4 °C on hold. Analysis of CREB gene expression in treated cells was performed by qRT-PCR on Light Cycler 96 instrument (Roche Diagnositics Ltd, Welwyn Garden City, UK) with AceQ qPCR SYBR Green Master Mix (Vazyme, Nanjing, CHN). Real-time PCR was performed in 20 μl reaction volumes containing SYBR Green Master Mix (10 μl), cDNA templates (100 ng), primers (2 μl) and RNase free water. Reaction conditions after initial denaturation at 95 °C for 10 min were: 40 cycles of 95 °C for 30 s and, 60 °C for 30 s, followed by 95 °C for 10 s, 65 °C for 60 s, 65 °C for 60 s, and 97 °C for 1 s. The original mRNA copies were evaluated by 2^−ΔΔCt^ method, and relative CREB mRNA concentrations were normalized to β-actin. The primers used were: *CREB1*, Forward 5′-ACTGTAACGGTGCCAACTCC-3′and Reverse 5′-GAATGGTAGTACCCGGCTGA-3′; *ACTB* (β-actin), Forward 5′-AGCGAGCATCCCCCAAAGTT-3′and Reverse 5′-GGGCACGAAGGCTCATCATT-3′.

### siRNA and transient transfection

Chemically synthesized BDNF siRNA targeting BDNF was purchased from Santa Cruz (USA). KGN cells were seeded at a density of 1 × 10^5^ cells per well in 6-well culture dishes with antibiotic-free DMEM/F12 medium supplemented with FBS. At 30–50% confluency, the cells were transfected with BDNF-siRNA, control siRNA, or control/BDNF siRNA plus FSH, for 7 h at 37 °C. After incubation, 1 ml DMEM/F12 medium was added to each well without removing the transfection mixture; the cells were incubated for an additional 24 h. BDNF level in KGN cells were assessed by Western blotting after transient transfection. Negative control siRNA was used to assess nonspecific gene-silencing effects.

### Statistic analysis

All statistical analyses were conducted with the SPSS 21.0 software package. One-way analysis of variance (ANOVA) was used for analysis, and differences between control and treatment groups were tested by Dunnett’s multiple comparison test. Data are mean ± SD from three independent trials; statistical significance was set at *P* < 0.05.
